# Round the Hospitals

**Published:** 1918-05-11

**Authors:** 


### ROUND THE HOSPITALS.

IT gives us great pleasure to publish this week, page 127, the third annual report of the Council of the College of Nursing and the Honorary Treasurer's report, together with the reports of the Scottish and Irish Boards. They constitute most interesting reading, contain many facts of importance, and testify to the splendid development of the College, and its acceptance by trained nurses as a source of strength. These reports must prove welcome to the nursing profession and to every knowledgeable person who can appreciate the remarkable position which the College of Nursing has already reached. The Treasurer's report, with the audited statements of account (which latter we have no space for this week), show that the Accumulated Fund now amounts to £8,692 10s. 10d., which has been invested in 5 per cent. War Loan and National Bonds. This amount is more than the total amount of fees received from members of the College for registration, inclusive of all expenses of management to date, which have been met by donations and interest on investments. It has always been the intention that the odd shilling of the Registration Fee should be used towards the expenses en-

Previous articles appeared Dec. 8, 15, 22, Jan. 5, 12, 26, Feb. 2, March 2, 9, 23, 30, and April 13, p. 33.

**ROUND THE HOSPITALS**—(*continued*).

tailed in the registration, including postage, stationery, etc. The accounts show, however, that the shillings in question have not been expended, but have been invested in the securities already mentioned. These facts put one more nail in the coffin containing the falsehoods and unfounded charges which the enemy, with untiring pertinacity, has endeavoured to get circulated through the Press with no appreciable success. We take this opportunity on behalf of more than 9,000 trained nurses who have become members of the College, who now constitute the most representative body of trained nurses in the Empire, to thank the Press throughout the country for the independence, impartiality, and help which editors of all shades of opinion have patriotically extended to the College of Nursing (Limited by Guarantee).

---

A CORRESPONDENT has handed us a letter, published, anonymously, from a Member of Parliament, which exhibits some curious misapprehensions and one good point. The good point is that the M.P. brushes aside the enemy's continuous plaint that gifts from grateful appreciators of the great sacrifices rendered by nurses, to the College Endowment Fund, are charity. No M.P. with any claim to represent anybody could do less than that, and retain his seat. Eleemosynary gifts and benevolent founders have been part of the life and backbone of important movements and institutions for generations. The misapprehensions are that M.P. appears to accept the enemy's falsehood which states that the sufferings, services, and rewards of military nurses, and nurses' sacrifices, sufferings, and service in the war are being made a lever by the College of Nursing (Limited by Guarantee) to get from the public, grateful for their great services, and from the officers and men who have profited by them, contributions which in part at any rate are intended to be devoted not to these nurses, or to nurses broken in the war, but to the establishment of a College for the manufacture of future nurses. We can assure M.P. that these statements are a myth so far as the College of Nursing is concerned, and that they ought to cover with confusion, as they have already covered with discredit, the interested persons who write, circulate, and utter misrepresentations and tarradilloes of this type.

---

AGAIN it is palpably untrue and without substance in fact that the College is exploiting, or ever has exploited, the sufferings and services of existing nurses for the benefit of those who are not now nurses, but who may in the future take up the profession. The College has made it perfectly clear that the welfare, protection, and advancement of trained nurses in this country must be incalculably benefited by the foundation of the College of Nursing and its endowment. The strongest appeal to those who have the means to contribute to an endowment fund is that the provision of £250,000 for this purpose will immeasurably strengthen the position of trained nurses in this country, and raise the standard and opportunities for trained nurses to a point, which must confer inestimable benefits on the working nurse of to-day and henceforward as the world progresses. A few persons for their own ends have circulated falsehoods concerning the exploitation of the sufferings, services, and rewards of military nurses, have molested members of the public attending places of amusement, and have generally set themselves to attempt to mislead public opinion by means which are as discreditable as they are unworthy of any woman, especially if that woman happens to be a partially trained or fully trained nurse. We have had the advantage of hearing all about the future of the Nation's Fund for Nurses, and are happy to testify that it is being organised so as to secure that the funds given to it will be invested in the names of trustees of repute, and that its administration will be in the hands of those who properly possess the public confidence, as well as the confidence and gratitude of British nurses of every shade of opinion.

---

THE College of Nursing is moving forward with lengthening strides in response to the ever-growing interest and support which trained nurses are giving to it. Every nurse who is a member of the College will surely book June 6, the date of its third Annual General Meeting, which has created a general wish amongst nurses that an opportunity should be given, under the auspices of the College, for members to meet and discuss matters of professional interest. To accomplish this end arrangements have been made whereby hospitality will be offered, as far as possible, to those members of the College coming from a distance to attend the Annual General Meeting. The details of these arrangements are now being settled, with the object of providing that this meeting shall be followed by a conference on June 7, and any member who has suggestions to make, or subjects for discussion to submit, should address them to the Secretary of the College of Nursing (Limited by Guarantee), 6 Vere Street, London, W.1. Every member, and indeed every trained nurse, should make it her business to realise and propagate the fact that hers is **not the College of Nursing (Limited), but the College of Nursing (Limited by Guarantee).** If it was **the College of Nursing (Limited),** it would be an ordinary financial company with shares, and so possessed of the objectionable features which the enemies of the College have falsely claimed and circulated continuously. In fact, the proprietors of the *British Journal of Nursing*, for instance, have turned themselves into a *limited company* of this type—*i.e.*, an ordinary trading company for profit, if possible. **The College of Nursing, on the contrary, is an association for mutual co-operation and help, not formed for purposes of profit and not requiring a trading capital, or having any capital at all. It is an association, not issuing any shares, but incorporated under the Companies Acts for the purpose of promoting the art and science of nursing.** Being an association not for profit, its full title is **The College of Nursing (Limited by Guarantee).** The use of the word

**ROUND THE HOSPITALS**—(*continued*).

**Limited,** in referring to the College of Nursing as its enemies have falsely promulgated, and all the prejudice and abuse they have wasted on this contention, have discredited nothing but themselves, who, by this abuse, have demonstrated to the business world and the public that, they are either impostors by intention, or simple ignoramuses biased by hostility and chagrin. Hence every trained nurse, and every member of the College especially, should realise the facts here set forth, and make it their business to enlighten their friends and all who may be interested in their profession, so that the solid foundation on which the College rests, and its consequent strength as an association **not for profit,** may be realised and understood. They should further note that the only difference between the Royal British Nurses' Association as an incorporated body, and the College of Nursing as an incorporated body, is that one is incorporated by charter and the other by Acts of Parliament administered by the Board of Trade.

Over 9,000 nurses have joined the College Register. Will not every one of them make it her business to do her utmost to recruit, at once, at least **o1e trained nurse** to apply for registration? for then the Register will contain 10,000 trained nurses by June 6, the date of the annual meeting. Indeed, it is manifest, if this plan is widely followed, that, those already on the Register can raise the number to 15,000, or more, by that date, if the work done by the readers of *The Nursing Mirror*, when the College was a baby, is borne in mind as a stimulus and encouragement to further and continuous effort. Every hospital staff might do much in this effort, if a move on is made now and continuously followed for four consecutive weeks.

The vitality of the College of Nursing in its new Manchester Centre was attested by the success of the recent public meeting in the out-patients' hall of the Royal Infirmary, at which Sir Arthur Stanley and Dame May Whitty were the principal speakers. The meeting resulted in the formation of a committee charged with the duty of organising the Nation's Fund for Nurses in the city, and an influential list of names was at once sent in of persons willing to help in this national work. The result can be in no way doubtful. Manchester is noted for its zeal in every department of philanthropic work, and has always dealt generously with the nurses essential to any scheme of social welfare. At this moment Manchester people are raising a fund of £20,000 in order to extend the operations of the Manchester and Salford Sick Poor and Private Nursing Institution, which for over fifty years has ministered to the sick in the city and surrounding district. It already maintains five homes and employs between seventy and eighty nurses. We have not the least doubt that the funds needed for this necessary branch of social service will be forthcoming, and that Manchester people will show their appreciation of nurses' work by subscribing liberally at the same time to the Nation's Fund. Meantime the Manchester Centre of the College is not neglecting its educational programme. The post-graduate lectures at the Royal Infirmary, given by Dr. Stopford, on the "Treatment of Gun-shot Wounds of Nerves," have been well attended, and those who were fortunate enough to be present learned some new and interesting features of the nursing treatment of these injuries.

The announcement of the formation of a Local Centre at Plymouth is particularly cheering for two reasons. In the first place the College meeting which took place in this great seaport (see The Hospital for March 30, page 557) resulted in the handing over of £80 to the committee of the British Women's Hospital towards the Nation's Fund for Nurses. In the second place, the nurses of the South Devon and East Cornwall Hospital have become so penetrated with a consciousness of what union will mean to the profession that they have subscribed among themselves the sum of £40 towards the formation of their Local Centre. With such a strong and enthusiastic nucleus of members the success of this Centre is assured. Both in Leeds and in Plymouth the effect produced by one meeting in starting fresh activities and in enlisting support shows very clearly what a priceless fund of hitherto unguessed-at zeal is ready to wake into life among nurses for self-governed associations. We are only at the beginning. Now that a strong impetus has been given to the organising ability of nurses displayed in furthering the interests of their fellow-nurses, there will be many surprises to follow. We look forward with hope and confidence to the achievements of the Local Centres.

The "serious deficiency of nursing members for naval and military hospitals" recently brought to public notice by the authorities requires, we fear, more than merely formal announcement if it is to be made good as speedily as circumstances demand. Recruiting for the Service needs to be carried out more systematically. The heads of schools and colleges should be approached, not with a purely official announcement, but with a more specialised appeal, setting out the various departments for which girls leaving school are required, and the preparation likely to be of use to them. Nearly every large educational establishment in the country would institute a holiday course of preparation for V.A.D. workers under a trained nurse if requested to do so. The sudden plunge at eighteen into hospital life, without the slightest preparation, is one which parents dread for their girls. If there were preparatory classes, with a short residential trial of nursing duties under trained nurses, with demonstrations which parents could attend if they desired, much enthusiasm would be roused, and the authorities would get as many girls as they wanted. There is still a great deal of prejudice to

**ROUND THE HOSPITALS**—*(continued)*.

break down in middle-class homes against the theory of hospital duties for young women, and eighteen is not always a very robust age. In short, preparation, and again preparation is what we would urge as the solution of the V.A.D. recruiting difficulty.

An excellent report comes to hand of the native nurses trained by the medical missions of the Church Missionary Society. Miss Tiffen, who has worked for sixteen years in one of these missions in Palestine, has recently given an address in Ipswich in which she stated that the native nurses had many of them remained at their posts at Gaza and elsewhere during the war, and that they had done fine work in helping to nurse the wounded. The British soldiers have spoken in the highest terms of their ministrations. Wherever native women are sympathetically handled and due regard is had to the limitations of their powers, great success attends the attempt to train them for service in the sick-room. But it is idle to expect to force them up to European pitch and put them through an elaborate curriculum at too early a stage in their development.

The forms which now have to be filled in at military hospitals showing how much margarine, meat, sugar, tea, etc., have been used per head in the establishment during the month are an admirable object-lesson to managers of all institutions. The old doctrine that economy was attainable by using "as little as possible" and leaving the result to chance has melted away under the pressure of war conditions. It is now an ascertained fact that economy results simply from knowledge of the amounts consumed per head, regularly recorded and compared one month with another. It gives a little more trouble, it is true, than using "as little as possible," but, on the other hand, the system introduces quite a new note of interest into domestic management. Its immediate effect is to set the housekeeper measuring out the quantity of tea and the bulk of margarine to be used in the preparation of the patients' teas, instead of leaving it to the discretion of the V.A.D. to use whatever she thinks fit. And without any ill-judged parsimony over the diet of the wounded, the result is very instructive.

The Burntwood Red Cross Hospital (Caterham) has brought out a timely Ready Reckoner for War Hospitals. It gives a compendium by means of which the maximum amount of each article of food allowed per patient can be reckoned in a few minutes for a period of from one to 100 weeks, and from one day to six days. The articles specified are meat, fish, bacon, bread, flour, sugar, margarine and suet, potatoes, fresh vegetables, cocoa, milk, syrup and jam, cereals, eggs, tea and coffee, cheese. The instructions for the use of the tables are simplicity itself. "When the number of daily counts has been ascertained for the week, always divide the counts by seven (the number of days in a week); having thus obtained the exact number of complete weeks and odd days, take the amounts [of any specified article] given in the Tables A and B [for weeks and days respectively] and add all together. The result will give the quantities, which should not be exceeded." By the use of these tables an infinity of troublesome calculations may be avoided, and the hospital accounts may be easily kept in such a way that it may be ascertained week by week whether the prescribed scale of rationing is being strictly adhered to. Matrons of civilian institutions, such as convalescent homes, receiving a preponderance of patients on ordinary diet would doubtless find the system tend to economy. For the military hospital this little ready reckoner is indispensable. It may be obtained from the Hon. Treasurer, Caterham Division, Surrey 88, Knaresdale, Caterham, price 1s. post free.

It is not quite certain that hospitals and other institutions are doing all that they might do to promote War Saving among members of the staff. Nothing is more gratifying than to note the cordial response which attends the effort of any public-spirited individual who will take up the duties of a War Savings Treasurer. Very little preliminary red-tape is necessary for the formation of a War Savings Association in an institution, and full particulars will be sent on application to the National War Savings Committee, Salisbury Square, E.C. 4. The essential thing is to appoint someone to collect every week such sums (which may be any multiple of sixpence) as the members may desire to pay in. When the spirit of the chase enters into the members, it is astonishing to find how much may be saved in little sums every week. Every walk undertaken to save omnibus fare is to the good. Every little indulgence forgone finds its immediate reward. And as the number of War Savings Certificates earned continues to increase, it is a cheering reflection that they will count as capital for the increase of a pension when the time comes that each 15s. 6d. has become a pound. Many Home sisters debarred from service abroad are earnestly desirous of "war work." To such we commend this vital and most interesting service for the State.

Only those on the spot fully recognised the difficulties which confronted the new matron of the Malton, Norton, and District Cottage Hospital, Yorkshire, when she took up her duties six months ago. In this period Miss H. Gardner has succeeded in the main in re-establishing the hospital's standard, and she deserves recognition for an uphill piece of work conscientiously attacked. Cottage hospital matronships are sometimes supposed to be "soft billets" by those who have not learnt that the smaller the institution the greater may be the scope for personal jealousies and all the obstructive forces which let and hinder efficient management. Cottage hospitals may be schools for saints as easily as homes of quiet.

☛ For the Shortage of Domestic Help, see p. 129; Nurses' Tribute Fund, Leicester Meeting, p. 130; Matrons' and Sisters' Appointments, p. vi.

